# A monoclonal antibody interferes with TIMP-2 binding and incapacitates the MMP-2-activating function of multifunctional, pro-tumorigenic MMP-14/MT1–MMP

**DOI:** 10.1038/oncsis.2013.44

**Published:** 2013-12-02

**Authors:** S A Shiryaev, A G Remacle, V S Golubkov, S Ingvarsen, A Porse, N Behrendt, P Cieplak, A Y Strongin

**Affiliations:** 1 Sanford-Burnham Medical Research Institute, La Jolla, CA, USA; 2 The Finsen Laboratory, Rigshospitalet/Biotech Research and Innovation Centre (BRIC), University of Copenhagen, Copenhagen, Denmark

**Keywords:** monoclonal antibody, TIMP-2, MT1–MMP, cancer, proteinase inhibitor

## Abstract

Matrix metalloproteinases (MMPs) and, especially membrane type 1 (MT1)-MMP/MMP-14, are promising drug targets in malignancies. In contrast with multiple small-molecule and protein pan-inhibitors of MT1–MMP cleavage activity, the murine 9E8 monoclonal antibody targets the MMP-2-activating function of cellular MT1–MMP alone, rather than the general proteolytic activity and the pro-migratory function of MT1–MMP. Furthermore, the antibody does not interact in any detectable manner with other members of the membrane type (MT)-MMP family. The mechanism of this selectivity remained unknown. Using mutagenesis, binding and activity assays, and modeling *in silico*, we have demonstrated that the 9E8 antibody recognizes the MT-loop structure, an eight residue insertion that is specific for MT–MMPs and that is distant from the MT1–MMP active site. The binding of the 9E8 antibody to the MT-loop, however, prevents tissue inhibitor of metalloproteinases-2 (TIMP-2) association with MT1–MMP. As a result, the 9E8 antibody incapacitates the TIMP-2-dependent MMP-2-activating function alone rather than the general enzymatic activity of human MT1–MMP. The specific function of the 9E8 antibody we determined directly supports an essential, albeit paradoxical, role of the protein inhibitor (TIMP-2) in MMP-2 activation via a unique membrane-tethered mechanism. In this mechanism, the formation of a tri-molecular MT1–MMP
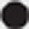
TIMP-2
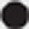
MMP-2 complex is required for both the capture of the soluble MMP-2 proenzyme by cells and then its well-controlled conversion into the mature MMP-2 enzyme. In sum, understanding of the structural requirements for the 9E8 antibody specificity may pave the way for the focused design of the inhibitory antibodies against other individual MMPs.

## Introduction

Matrix metalloproteinases (MMPs) belong to a zinc endopeptidase, metzincin superfamily that is distinguished from other proteases by the presence of a conserved HEXXHXXGXX(H/D) sequence motif with three His residues that chelate the active site zinc.^1^ In humans, MMPs are represented by 24 enzymes, which share several functional domains.^2^ Membrane type (MT) MMPs are distinguished from soluble MMPs by an additional transmembrane domain and a cytoplasmic tail (MT1–MMP/MMP-14, MT2–MMP/MMP-15, MT3-MMP/MMP-16 and MT5–MMP/MMP-24), whereas MT4–MMP/MMP-17 and MT6–MMP/MMP-25 are attached to the cell membrane via a glycosylphosphatidyl inositol anchor. MMPs are synthesized as latent zymogens that require proteolytic activation to remove the N-terminal inhibitory prodomain. Arguably, pro-invasive membrane type 1 (MT1)-MMP is the most relevant MMP in cancer and a promising drug target in malignancies.^3, 4, 5^ MT1–MMP knockout has a profound effect: null mice develop dwarfism, bone malformations and die before adulthood, whereas knockouts in other MMP genes in mice do not elicit an easily recognized phenotype.^6^

Once activated, MMPs may be inhibited by tissue inhibitors of MMPs (tissue inhibitor of metalloproteinase (TIMP)-1, -2, -3 and -4).^7^ The MMP/TIMP balance is a major factor in the regulation of the net proteolytic activity of MMPs. Structurally, TIMPs contain two domains. The inhibitory N-terminal domain binds the MMP catalytic domain, blocking access of substrates to the active site. The C-terminal domain of TIMP-1 and TIMP-2 binds to the hemopexin domain of the proenzymes of MMP-9 and MMP-2, respectively.^8, 9^

Membrane-tethered MT1–MMP is a key enzyme in the activation of soluble MMP-2.^10, 11^ Cellular MT1–MMP, however, performs multiple pericellular cleavage functions, which are additional to and distinct from activation of MMP-2 and MMP-13 and which include degradation of the extracellular matrix proteins, including collagen, and proteolysis of a significant number of cell adhesion and signaling receptors.^12^ Because TIMPs and active site-targeting small-molecule inhibitors fully inactivate the catalytic activity, their use does not allow us to discriminate the individual functions of MT1–MMP. As a result, the importance of both MMP-2 activation and the active MMP-2 enzyme itself in the net proteolytic function of MT1–MMP in normal development and in disease remains unidentified.

Recently, a specific monoclonal antibody 9E8 (mAb 9E8) against MT1–MMP has been raised in an MT1–MMP null mouse.^13^ In contrast to other reported function-blocking mAbs against MT1–MMP, which became available recently, including DX2400,^14, 15, 16, 17^ mAb 9E8 targets a single function of multifunctional cellular human MT1–MMP: its ability to activate the MMP-2 proenzyme. Here, using antibody-peptide binding assays combined with mutagenesis, activity and cellular assays, and structural modeling, we identified the structural requirements for the unique inhibitory activity of mAb 9E8.

## Results

### Antibody-peptide binding

**Figure 1:**
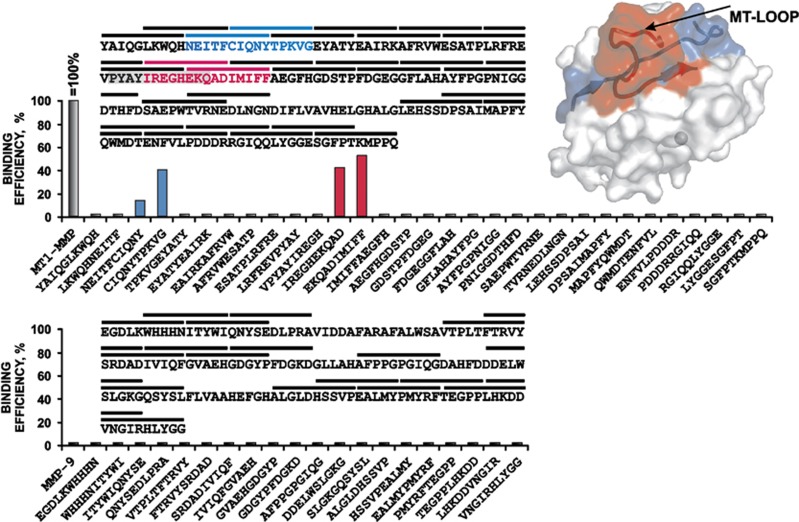
Binding of mAb 9E8 to peptides derived from the catalytic domain of MT1–MMP. Top, Left, the sequence of the catalytic domain of MT1–MMP. The lines above the sequence mark the 10-residue peptides overlapping by 5 residues. The N- and C-terminal binding sequences (identified for mAb 9E8) are shown in blue and red, respectively. The MT-loop sequence is shaded gray. Right, the structure of the catalytic domain (PDB accession 1BQQ) showing the location of the 9E8-binding peptide sequences. Bottom, the efficiency of the 9E8 binding to the individual MMP-9 peptides and the catalytic domain of MMP-9 relative to that of the purified catalytic domain of MT1-MMP (=100%, 1 μg). Active site zinc, gray sphere.

First, to identify the potential antibody-binding sequences in MT1–MMP, we allowed mAb 9E8 to bind synthetic peptides immobilized on a nitrocellulose membrane. The 10-residue peptides overlapping by five residues represented the solvent-exposed molecular surface of the MT1–MMP catalytic domain. In these assays using the mAb 9E8, a significant level of reactivity was recorded with the peptides NEITFCIQNY, CIQNYTPKVG, IREGHEKQAD and EKQADIMIFF (an overlap is underlined) suggesting that the antibody recognizes a structural region that is distinct from the MT1–MMP active site. The potential binding site of mAb 98E included the MT-loop represented by the C-terminal PYAYIREGHEKQ 163–174 sequence in MT1–MMP and the additional, N-terminal NEITFCIQNYTPKVG 122–136 sequence region (Figure 1).

No interaction of mAb 9E8 was detected under the similar experimental conditions with either the catalytic domain of MMP-9 (that does not exhibit the MT-loop) or any of the 10-residue control peptides derived from the molecular surface of this catalytic domain (Figure 1). Similarly, as judged by the results of Western blotting and fluorescent peptide cleavage assays, mAb 9E8 neither binds nor inhibits the MMP-2, MT2–MMP/MMP-15, MT3-MMP/MMP-16, MT4–MMP/MMP-17, MT5–MMP/MMP-24 and MT6–MMP/MMP-25 catalytic domain constructs (not shown).

### Inhibitory assays

**Figure 2:**
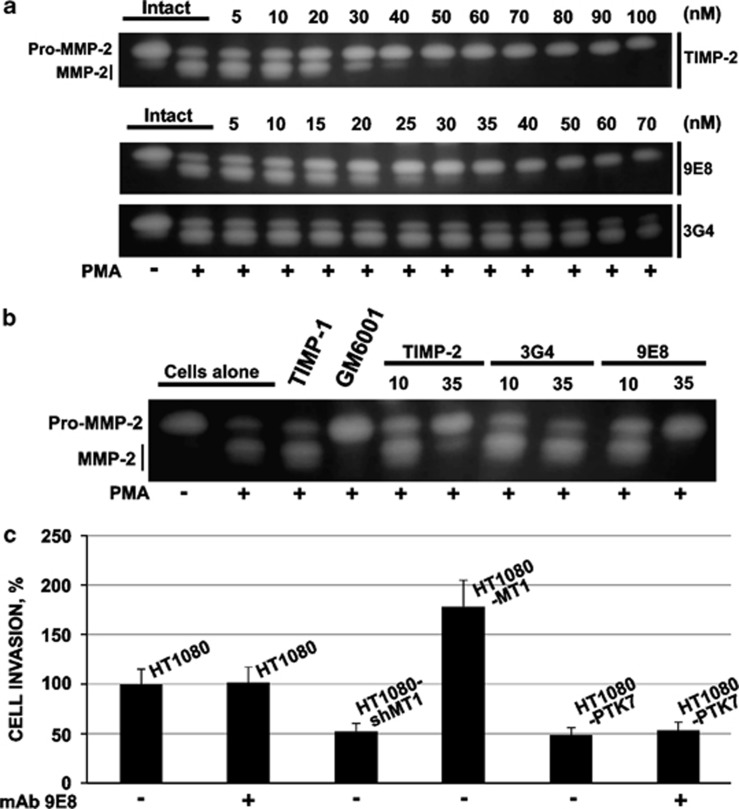
mab 9E8 inhibits MMP-2 activation by MT1–MMP. (**a**) Gelatin zymography of MMP-2. Where indicated, HT1080 cells were stimulated with phorbol 12-myristate 13-acetate and coincubated with the increasing concentrations of TIMP-2 and mAbs 9E8 and 3G4. (**b**) Gelatin zymography of MMP-2. Where indicated, HT1080 cells were stimulated with phorbol 12-myristate 13-acetate and coincubated with TIMP-1 (100 nM), GM6001 (10 μM), TIMP-2 and mAbs 9E8 and 3G4. Numbers show the concentrations in nM. (**c**) Cell invasion through the collagen layer. HT1080, the wild-type HT1080 cells. HT1080-shMT1, the cells in which MT1–MMP was silenced by small hairpin RNA (shRNA). HT1080-MT1, the cells, which stably overexpressed the full-length human MT1–MMP. HT1080-protein tyrosine 7 (PTK7), the cells, which stably overexpressed the anti-migratory full-length PTK7 construct.^20, 21, 22^ Cell invasion levels were calculated relative to the untreated wild-type HT1080 cells (=100%). Where indicated, mAb 9E8 (100 nM) was added to both inner and outer chambers of the transwell inserts.

Exogenous TIMP-2 and mAb 9E8 at a 25–35 nM range are similarly potent in inhibiting MT1–MMP-dependent activation of MMP-2 in phorbol 12-myristate 13-acetate-stimulated HT1080 cells, whereas TIMP-1 (a known inefficient inhibitor of MT1–MMP) and the non-inhibitory mAb 3G4^18^ were without effect (Figures 2a and b). These data correlate well with the known binding affinities of TIMP-2 and mAb 9E8 with MT1–MMP, both of which are in a sub-nanomolar range.^13, 19^ Even at the very high, 100 nM, concentration, mAb 9E8 had no inhibitory effect on invasion of the wild-type HT1080 cells and HT1080 cells, which have been stably transfected with the anti-migratory full-length protein tyrosine kinase-7 (PTK7) construct. Cells, which stably overexpressed MT1–MMP and, additionally, cells in which MT1–MMP was silenced by the highly efficient and proven small hairpin RNA construct were used as a positive and negative control in these experiments, respectively (Figure 2c).^20, 21, 22^

**Figure 3:**
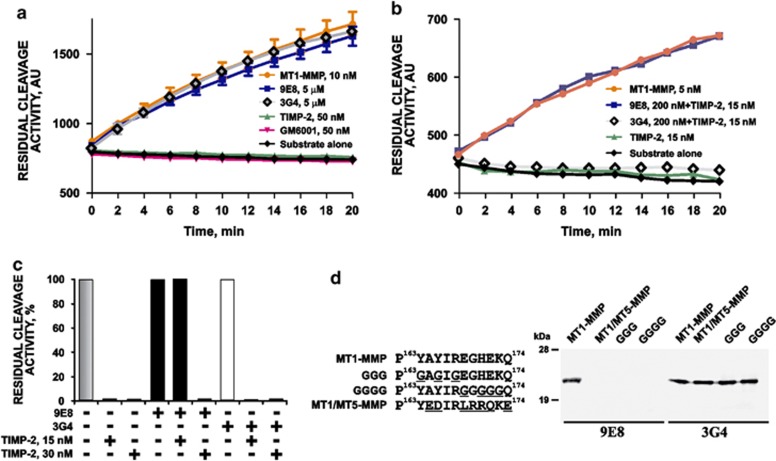
mAb 9E8 does not inhibit the general proteolytic activity of MT1–MMP. (**a**) Residual activity of MT1–MMP (10 nM) against the MCA-PLGL-Dpa-AR-NH_2_ substrate. Where indicated, TIMP-2, GM6001 or mAbs 9E8 and 3G4 were added to the reactions. (**b**) Residual activity of MT1–MMP (5 nM) against the MCA-PLGL-Dpa-AR-NH_2_ substrate. Where indicated, TIMP-2 alone or TIMP-2 jointly with mAb 9E8 or mAb 3G4 were added to the reactions. (**c**) Residual activity of MT1-MMP (5 nM) against the MCA-PLGL-Dpa-AR-NH_2_ substrate. The indicated concentrations of TIMP-2 and mAbs 9E8 or 3G4 were added to the reactions. (**d**) Mutations in the MT-loop inactivate the ability of mAb 9E8 to bind MT1–MMP. The original MT1-MMP catalytic domain and the MT1/MT5–MMP, GGG and GGGG mutant constructs (the sequences are on the left) were analyzed by western blotting with mAbs 9E8 and 3G4. AU, arbitrary unit.

Similarly with the non-inhibitory mAb 3G4, mAb 9E8 at a 1:500 enzyme-inhibitor molar ratio, was incapable of affecting the cleavage activity of MT1–MMP against the peptide substrate. In contrast, active site-targeting GM6001 and TIMP-2, even at a much lower excess (at a 1:5 enzyme-inhibitor molar ratio), readily suppressed the peptide-cleaving activity of MT1–MMP (Figure 3a).

The presence of mAb 3G4 (200 nM) in the peptide cleavage reactions did not affect the ability of TIMP-2 (15–30 nM; a 1:3-1:6 enzyme inhibitor molar ratio) to fully block the peptide-cleaving activity of MT1–MMP (Figure 3b). In contrast, the inhibitory activity of TIMP-2 (15 nM) against MT1–MMP was completely repressed in the presence of 200 nM mAb 9E8. However, an additional twofold increase in TIMP-2 (30 nM) completely abolished the effect of mAb 9E8 and the full inhibition of the proteolytic activity of MT1–MMP was observed, suggesting a direct competition between TIMP-2 and mAb 9E8 (but not mAb 3G4) for the binding with MT1–MMP (Figure 3c).

### Mutagenesis of the MT-loop

To corroborate our epitope mapping results, we mutated the P^163^YAYIREGHEKQ^174^ MT-loop sequence in MT1–MMP (Figure 3d). In the GGG mutant, Gly residues substituted for Tyr164, Tyr166 and Arg168 in the N-terminal portion of the MT-loop. In the GGGG mutant, Gly residues substituted for Glu169, His171, Glu172 and Lys173 in the C-terminal portion of the MT-loop. In the MT1/MT5–MMP mutant, the MT-loop sequence from MT5–MMP substituted for the MT1–MMP sequence. The ability of mAbs 3G4 and 9E8 to interact with the purified mutant samples was determined using western blotting with the respective antibodies. The GGG, GGGG and MT1/MT5–MMP mutants were capable of interacting with mAb 3G4 as efficiently as the original MT1–MMP construct. In turn, mAb 9E8 recognized the wild-type construct alone but neither of the mutants. On the basis of these data and in agreement with our antibody-peptide binding assays, we concluded that the P^163^YAYIREGHEKQ^174^ MT-loop sequence was essential for the interaction with mAb 9E8. In agreement, mAb 9E8 equally efficiently interacted with human and murine MT1–MMP species, both of which exhibit the identical 122–136 (NEITFCIQNYTPKVG) and 163–174 (PYAYIREGHEKQ) epitope sequences.

### Modeling of the MT1–MMP complex with TIMP-2 and the antibody

**Figure 4:**
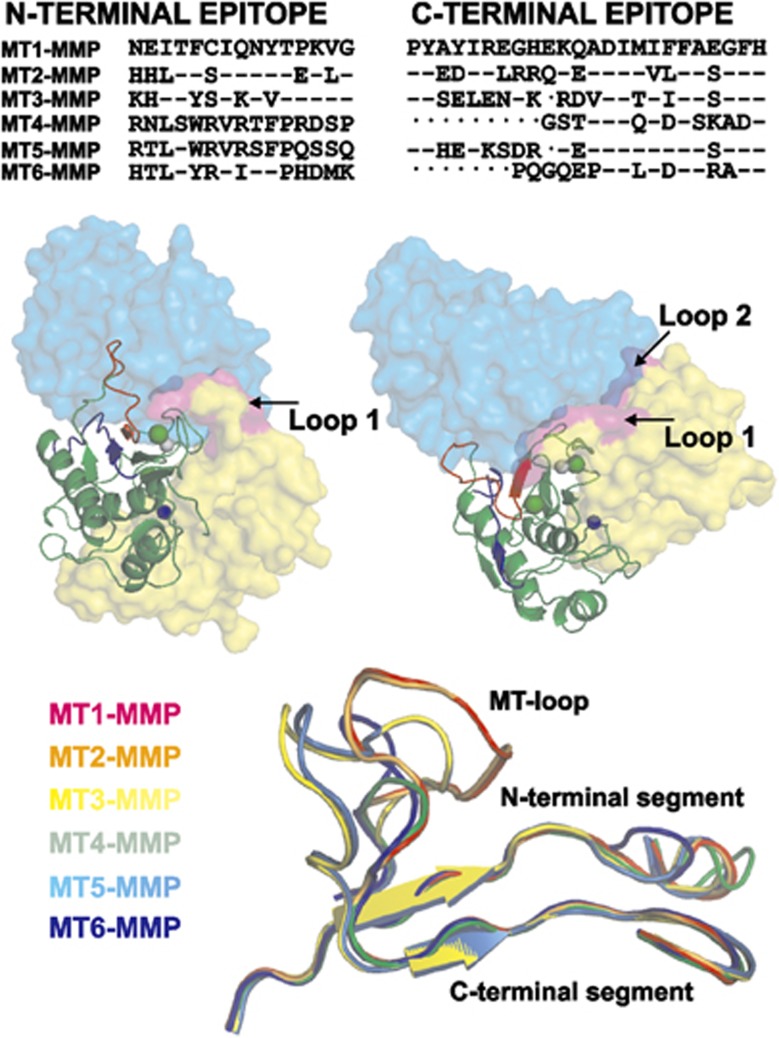
mAb 9E8 competes with TIMP-2 binding to the catalytic domain of MT1–MMP. Top, the alignment of the identified N- and C-terminal epitope sequences in the MT–MMP subfamily. ‘–‘ and ‘.' symbols indicate identity and deletion, respectively. Middle, two views of the predicted MT1–MMP complexes with TIMP-2 (yellow) and the antibody (blue). MT1–MMP is shown as cartoon with the N- and C-terminal 9E8-binding regions in blue and red, respectively. Active site zinc, blue sphere. Note the putative penetration (pink) of the antibody moiety into the space occupied by TIMP-2 (predominantly, loops 1 and 2 of TIMP-2). Bottom, structural alignment of the N- and C-terminal regions in the MT–MMP subfamily. The N- and C-terminal regions were structurally aligned using FATCAT.^37^ The MT1–MMP and MT3-MMP structures were adopted from the PDB accession 1BQQ and accession 1RM8 templates, respectively.^38^ The structures of MT2–MMP, MT4–MMP and MT6–MMP and of MT5–MMP were modeled using the 1BQQ and 1RM8 templates, respectively.

To estimate the space occupied by the 9E8 antibody and TIMP-2, we used the three dimensional structure of the MT1–MMP
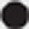
TIMP-2 complex (Protein Data Bank (PDB) accession 1BQQ) and the anti-Ras antibody (PDB accession 2UZI) as templates. We then modeled the putative tri-molecular complex involving TIMP-2, MT1–MMP and the variable regions of the light and heavy chains of the antibody. In the modeled structures, the complementarity determining regions of the antibody were positioned proximal to the MT-loop of MT1–MMP. According to our modeling, there is a penetration of the antibody moiety into the space occupied by the loops 1 and 2 of TIMP-2 (Figure 4). These results correlate well with the competition between TIMP-2 and mAb 9E8 we observed in our inhibitory assays. The MT-loop structure is present in all members of the MT–MMP family. However, both the size and the sequence of the MT-loop are different among MT–MMPs (Figure 4), the features that could be used for the design of the specific antibodies to the individual members of the MT–MMP family.

## Discussion

Recently, a unique mAb 98E that targets a single function of the multifunctional cellular MT1–MMP enzyme, an archetype membrane-anchored MMP, has been developed.^13^ Specifically, mAb 9E8 blocks the ability of cellular MT1–MMP to activate the MMP-2 proenzyme without interfering with the general proteolytic activity and the pro-migratory function of MT1–MMP. The inhibitory mechanism of this antibody, however, remained incompletely understood. Our study was focused on shedding additional light on both the structural requirements that are essential for the uniqueness of the mAb 9E8 mode of action and the mechanism of MMP-2 activation.

Paradoxically, our data imply that mAb 9E8 achieves its inhibition of the MMP-2-activating activity of MT1–MMP through the interactions with the structures, which are both distinct and distant from the catalytic and substrate-binding sites of the protease. These interactions involve the structures, which include the MT-loop and which are localized on the opposite side of the MT1–MMP molecule relative to the catalytic cleft. As a result, the interactions with these non-active site structures do not affect the enzyme catalysis performed by the active site. Our current data directly correlate with the results of others^23^ who demonstrated the importance of the MT-loop in the ability of cellular MT1–MMP to activate the MMP-2 proenzyme. Because of the unique sequence of the mAb 9E8 epitopes, this antibody is highly selective for MT1–MMP. As a result, mAb 9E8 does not interact with any additional individual MMP species, including the individual MT–MMPs, all of which exhibit the distinct peptide sequence in the regions, which correspond to the mAb 9E8 epitopes (Figure 4).

According to our results, the distinctiveness of the mAb 9E8 inhibitory mechanism is linked to the uniqueness of the MMP-2 activation mechanism.^10, 11, 24^ In this activation mechanism, cell-surface MT1–MMP acts as a receptor for TIMP-2. TIMP-2 binds via its N-terminal domain to the MT1–MMP catalytic domain. The TIMP-2's C-terminal domain then binds the hemopexin domain of proMMP-2 resulting in the formation of a tri-molecular MT1–MMP
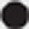
TIMP-2
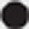
proMMP-2 complex. A second, TIMP-2-free MT1–MMP molecule, which is close to the complex, then cleaves the propeptide of the MMP-2 zymogen, generating a MMP-2 intermediate species, which then converts to the MMP-2 mature enzyme by self-proteolysis. The absence of TIMP-2 makes the MT1–MMP-dependent activation of MMP-2 impossible. Our experimental assays combined with structural modeling based on the available atomic resolution structures of MT1–MMP, TIMP-2 and multiple antibodies suggest that the binding of mAb 9E8 to the catalytic domain of MT1–MMP creates steric hindrance for the binding of TIMP-2 and *vice versa*. In other words, TIMP-2 and mAb 9E8 compete with each other for the binding with MT1–MMP.

Because the antibody excess blocks the binding of TIMP-2 via its N-terminal domain to the MT1–MMP catalytic domain, it is now not surprising that the antibody represses the MMP-2-activating capacity of MT1–MMP alone rather than the general cleavage activity and pro-migratory function of MT1–MMP. Our earlier experiments, in which we measured the ability of the wild-type (MT1–MMP^+/+^) and MT1–MMP^−/−^ fibroblasts to release ^125^I-labeled type I collagen in the presence and in the absence of the mAb 9E8, demonstrated that the antibody did not uncover any additional proteolytic activity of cellular MT1–MMP.^13^ In agreement, mAb 9E8 did not affect MT1–MMP-dependent invasion of human fibrosarcoma HT1080 cells (a widely accepted cell system for the MT1–MMP studies) through the collagen layer.

According to the results by us and other, cellular active MT1–MMP is a short-lived enzyme that is either self-proteolyzed in the absence of TIMP-2 or inhibited by TIMP-2 and, therefore, the functional activity of MT1–MMP is tightly controlled at the cell surface.^25, 26, 27, 28^ Because of these unconventional characteristics of cellular MT1–MMP and based on the results of our invasion assays, which employed mAb 9E8, it is highly likely, as a result, that the mAb 9E8 does not uncover the action of TIMP-2-inhibitable MT1–MMP activity against any substrates that are additional to and distinct from the MMP-2 proenzyme. Overall, the data directly support and highlight the essential, unique role of TIMP-2 in the cell-surface, MT1–MMP/TIMP-2-dependent mechanism of MMP-2 activation.^11^

The availability of mAb 9E8, a highly valuable molecular tool, makes it possible, for the first time, to selectively evaluate the importance of both MMP-2 activation and the active MMP-2 enzyme itself in the net proteolytic function of MT1–MMP. In addition, we suggest that the understanding of structural requirements for the mAb 9E8 specificity paves the way for the focused design of the antibodies against other MMPs.

## Materials and methods

### Reagents

The reagents were purchased from Sigma-Aldrich (St Louis, MO, USA) unless indicated otherwise. Fluorescent peptide substrate MCA-PLGL-Dpa-AR-NH_2_ was purchased from R&D Systems (Minneapolis, MN, USA). Murine monoclonal non-inhibitory mAb 3G4 against the catalytic domain of MT1–MMP and a hydroxamate inhibitor GM6001 were from EMD Millipore (Temecula, CA, USA). TIMP-1 was obtained from Invitrogen (Carlsbad, CA, USA). The murine mAb 9E8 antibody was described earlier.^13^ Peptides were synthesized by Spyder Institute (Prague, Czech Republic). Original human fibrosarcoma HT1080 cells (HT1080 cells) were from ATCC (Manassas, VA, USA). HT1080 cells stably transfected with the full-length MT1–MMP (HT1080-MT1 cells), the MT1–MMP-silencing small hairpin RNA construct (HT1080-shMT1 cells) and the full-length PTK7-FLAG construct (HT1080-PTK7 cells) were characterized earlier.^20, 21, 22^

### Enzyme cloning, expression and purification

The cloning, expression and purification of the wild-type MT1–MMP catalytic domain (residues 112–285), the catalytically inactive E240A MT1–MMP mutant and recombinant human TIMP-2 (C-terminally tagged with a 6xHis tag) were described previously.^29^ The purified samples (the purity >95%) were used in our subsequent studies. The concentration of the catalytically active MMPs we used in our study, including MT1–MMP, was measured by titration of the MMP samples against a standard GM6001 solution of known concentration and then measuring the residual activity against the MCA-PLGL-Dpa-AR-NH_2_ substrate. The steady-state rate of the substrate cleavage by MMP was plotted as a function of inhibitor concentration and fitted with the equation V=SA(E_0_−0.5{(E_0_+I+*K*_*i*_)−[(E_0_+I+*K*_*i*_)^2^−4E_0_I]^0.5^}), where V is the steady-state rate of substrate hydrolysis, *SA* is specific activity (rate per unit of enzyme concentration), E_0_ is enzyme concentration. I is inhibitor concentration and *K*_*i*_ is the dissociation constant of the enzyme-inhibitor complex.^30, 31^

### The MT-loop MT1–MMP mutants

The MT-loop mutants were obtained by PCR using the E240A mutant sequence as a template. The mutant constructs were then re-cloned into the pET101 vector (Invitrogen), expressed in *Escherichia coli*, purified from inclusion bodies using metal-chelating chromatography and refolded to restore the native conformation. A typical yield of the purified MT1–MMP constructs was 1 mg from 10 mg inclusion bodies. The ability of the mutants to interact with the antibodies was tested using Western blotting. Following the transfer of the separated proteins to a membrane, the latter was blocked using phosphate-buffered saline-1% casein and incubated with the intact mAbs 9E8 or 3G4 followed by the donkey anti-mouse horseradish peroxidase-conjugated immunoglobulin G (Jackson ImmunoResearch, West Grove, PA, USA) and a TMB/M substrate (SurModics, Eden Prairie, MN, USA).

### Protease activity assay

The cleavage assays were performed in triplicate in wells of a 96-well plate using the purified wild-type catalytic domain of MT1–MMP (10 nM) and the fluorescent peptide MCA-PLGL-Dpa-AR-NH_2_ substrate in 0.2 ml 50 mM HEPES, pH 6.8, containing 1 mM CaCl_2_, 0.5 mM MgCl_2_ and 10 μM ZnCl_2_. Where indicated, before the cleavage reactions the indicated concentrations of TIMP-2 alone or jointly with mAbs 9E8 or 3G4 were coincubated for 30 min at 20 °C with MT1–MMP samples. Initial reaction velocities were monitored continuously at *λ*_ex_=320 nm and *λ*_em_=400 nm on a fluorescence spectrophotometer.

### Antibody-peptide binding assay

Using the MT1–MMP
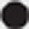
TIMP-2 complex structure (PDB accession 1BQQ) as a guide, we synthesized the 31 10-residue long peptides overlapping by 5 residues (YAIQGLKWQH, LKWQHNEITF, NEITFCIQNY, CIQNYTPKVG, TPKVGEYATY, EYATYEAIRK, EAIRKAFRVW, AFRVWESATP, ESATPLRFRE, LRFREVPYAY, VPYAYIREGH, IREGHEKQAD, EKQADIMIFF, IMIFFAEGFH, AEGFHGDSTP, GDSTPFDGEG, FDGEGGFLAH, GFLAHAYFPG, AYFPGPNIGG, PNIGGDTHFD, SAEPWTVRNE, TVRNEDLNGN, LEHSSDPSAI, DPSAIMAPFY, MAPFYQWMDT, QWMDTENFVL, ENFVLPDDDR, PDDDRRGIQQ, RGIQQLYGGE, LYGGESGFPT and SGFPTKMPPQ). The peptides, when combined, represented the molecular surface of the catalytic domain of human MT1–MMP. Peptides (1 μg each) were spotted on a nitrocellulose membrane (Bio-Rad). The membrane was blocked using phosphate-buffered saline-1% casein (30 min, 20 °C), incubated for 2 h with mAbs 3G4 and 9E8 (0.5 μg/ml each), extensively washed in phosphate-buffered saline-0.05% Tween-20 and then incubated for 1 h with the donkey anti-mouse horseradish peroxidase-conjugated immunoglobulin G. The immunoreactive peptide spots were visualized using a TMB/M substrate. As a control, we also synthesized and tested 20 ten-residue peptides overlapping by 5 residues (EGDLKWHHHN, WHHHNITYWI, ITYWIQNYSE, QNYSEDLPRA, VTPLTFTRVY, FTRVYSRDAD, SRDADIVIQF, IVIQFGVAEH, GVAEHGDGYP, GDGYPFDGKD, AFPPGPGIQG, DDELWSLGKG, SLGKGQSYSL, ALGLDHSSVP, HSSVPEALMY, EALMYPMYRF, PMYRFTEGPP, TEGPPLHKDD, LHKDDVNGIR and VNGIRHLYGG), which, when combined, represented a significant portion of the molecular surface of the human MMP-9 catalytic domain (PDB accession 1GKC).^32^ To calculate the intensity of the reactive spots, the images were scanned and digitized.

### MMP-2 activation assay

The status of MMP-2 was analyzed using gelatin zymography of the serum-free medium aliquots (15 μl). Where indicated, fibrosarcoma HT1080 cells (1 × 10^5^ seeded in wells of a 48-well plate) were stimulated for 24 h with phorbol 12-myristate 13-acetate (50 ng/ml) alone and also in the presence of GM6001 (10 μM), TIMP-1 (100 nM), TIMP-2 (5–100 nM) and mAbs 3G4 or 9E8 (5–70 nM each).

### Cell invasion assay

Assays were performed in wells of a 24-well, 8 μm pore size Transwell plate (Corning, Corning, NY, USA). A 6.5 mm insert membrane was coated with 0.1 ml rat tail type I collagen (0. 1 mg/ml; BD Biosciences, Franklin Lakes, NJ, USA) and then air dried for 16 h. The collagen coating was rehydrated for 1 h in 0.1 ml Dulbecco's Modified Eagle medium. The inner chamber contained 0.6 ml Dulbecco's Modified Eagle medium–10% fetal bovine serum as a chemoattractant. Where indicated, mAb 9E8 (100 nM) was added to both inner and outer chambers. Cells (1 × 10^5^ in 0.1 ml serum-free Dulbecco's Modified Eagle medium) were allowed to migrate for 3.5 h at 37 °C in a CO_2_ incubator. The cells remaining on the top surface of the membrane were removed with a cotton swab. The cells on the bottom surface of the membrane were fixed and stained for 10 min using 0.5 ml 0.2% crystal violet in 20% methanol. The incorporated dye was extracted using 0. 3 ml 1% SDS and the A_570_ was measured using a plate reader. Data are means ±s.e. from three individual experiments performed in triplicate. Cell invasion levels were calculated relative to the untreated wild-type HT1080 cells (=100%).

### Molecular modeling

In our modeling studies, we used the structures of the anti-Ras antibody (PDB accession 2UZI)^33^ and the catalytic domain of MT1–MMP complexed with TIMP-2 (PDB accession 1BQQ).^34^ To estimate the space occupied by the antibody, the putative complex of the MT1–MMP catalytic domain with mAb 9E8 was modeled using ZDOCK,^35^ and 1BQQ and 2UZI as templates. Because anti-MT1–MMP mAbs 9E8 and DX2400^14, 15^ bind the similar regions of MT1–MMP, we also replaced *in silico*, using Modeller,^36^ the residue positions in the complementarity determining regions in the light and heavy chains (complementarity determining region-L-1, 2, 3 and complementarity determining region-H-1, 2, 3, respectively) in 2UZI using the respective residues in DX2400. These substitutions, however, did not significantly affect the size and the global fold of the modified 2UZI in its complex with MT1–MMP (not shown).
